# The research focus and development trend of art therapy in Chinese education since the 21st century

**DOI:** 10.3389/fpsyg.2022.1002504

**Published:** 2022-12-15

**Authors:** Yingying Cui, Fenping Wang

**Affiliations:** ^1^Moscow Art School, Weinan Normal University, Weinan, China; ^2^Shaanxi Normal University, Xi’an, China

**Keywords:** art therapy, special education, health education, group painting therapy, mandala painting therapy

## Abstract

Since the 21st century, art therapy has made great progress and development in China’s education. To find out the relationship between art therapy and special children, hundreds of related literatures were analyzed using co-word analysis software, which shows that art therapy is increasingly important in special children and their education. Expressive art therapy has a good development prospect in college students’ mental health education and group counseling. The connotation of group painting therapy is constantly enriched. The theme research of mandala painting therapy is closely related, and painting art therapy focuses on the psychological counseling research of different groups, but the two are still in the marginal position in the whole research, and have not been paid attention to. Therefore, in the future research, firstly, we should continue to strengthen the research of art therapy and expressive art therapy in special education and individual mental health education; second, further broaden the depth and breadth of group painting therapy; third, strengthen the study of mandala painting therapy and painting art therapy.

## Introduction

Some introverted individuals or special groups are not good at expressing their emotions verbally or choose to keep their innermost emotions hidden. However, when emotions are not released or expressed for a long period of time, mental illness can be triggered. Art therapy can help individuals to express their emotions and feelings through external art forms such as painting and shaping. Art therapy is a therapeutic approach (Kaimal and Arslanbek, 2020), with the assistance of an art therapy, the individual engages in visual expression of mental images through painting, shaping and other forms of art, through which unexpressed thoughts and emotions are expressed outwardly. The mental images expressed and presented have a therapeutic and diagnostic function, providing an indicator of how the therapist and client are processing during therapy. During therapy, the client’s emotions are often included in the artwork and are processed and resolved within the therapeutic relationship. Art therapy was introduced by British artist Adrian Hill in 1942 and it became a stand-alone therapy in the 1960s. In the 1980s, it was introduced into the country, and its therapeutic effects are good for special groups with limited verbal and emotional expression (Richard et al., 2015). Since the 21st century, more and more attention has been paid to quality education (Crissien-Borrero et al., 2019), with its emphasis on the all-round development of students’ morality, intellect, physique, esthetics and labor and on mental health education, which makes the art therapy increasingly important in education (Haeyen and Staal, 2021). In this context, it is important to explore the hotspots of art therapy in China’s education since the 21st century and to clarify the direction of development of art therapy in China’s education. In order to present the research results of art therapy in education in China since the 21st century in a more objective and intuitive way, and to provide knowledge support for the future research of art therapy in education in China, this study intends to draw a knowledge map of the hotspots of art therapy research in education in China since the 21st century with the help of scientific knowledge mapping technology, and to analyze the hotspots and trends of art therapy research accordingly, in order to provide some reference and guidance for the development of art therapy in education in China. This study aims to provide some reference and guidance for the development of art therapy in education in China.

## Sources of information and research methodology

In this study, in terms of data collection, we searched hundreds of literatures on art therapy and painting therapy. In terms of data consolidation, data statistics and data analysis, we adopt the bibliometric analysis to show the hot spots and development trends in art therapy.

### Sources of information

A precise search was conducted in the China National Knowledge Infrastructure (CNKI) journal database using the subject terms “art therapy” AND “painting therapy.” We searched relevant articles from 2001 to 2021 and got 738 articles. Excluding non-standardized and non-subject related literature such as conference proceedings, announcements and school profiles, 462 articles were identified as valid. The key words in the valid literature were standardized, for example, the terms “group painting therapy” and “group drawing treatment” were combined into “group painting therapy.”

### Research tools

This study adopts the bibliometric analysis to show the hot spots and development trends in art therapy and use Bicomb 2.0 co-word analysis software and SPSS23, in which Bicomb 2.0 co-word analysis software can analyze the data in terms of core authors, main research institutions and so on. It can also visualize the most cutting-edge areas of educational research through images, revealing an overview of the development of educational research at different levels, allowing researchers to take a comprehensive look at the structure of the field of educational research and information on research hotspots and priorities.

### Research process

Firstly, Bicomb 2.0 software was used to extract high-frequency keywords and generate a word-part matrix. Secondly, SPSS23 was used to cluster the word-part matrix to generate a similarity matrix and a dendrogram. In addition, the similarity matrix was used to conduct a multi-dimensional scale analysis and combined with the dendrogram to draw a knowledge map of research hotspots. Finally, the knowledge map was interpreted and analyzed for content.

## Findings of the study

### High frequency keyword statistics and analysis

The keywords of a paper are the words used by the author to precisely express the central idea of the paper. By analyzing the keywords of a paper, one can get a general idea of the topic of the paper and the author’s core ideas. Therefore, the keyword count of the selected papers was conducted by Bicomb 2.0 co-word analysis software. Four hundred and sixty-two papers with a total of 1,688 keywords were standardized and analyzed for word frequency statistics. The keyword threshold was determined according to the Price formula *M* = 0.749, where M represents the high frequency threshold and N_max_ represents the highest value of the frequency of literature cited (Yang et al., 2019). The highest value of literature cited frequency in the selected literature is 128, i.e., N_max_ = 128. Then, the formula was used to calculate *M* = 8.464, so 8 was determined as the minimum frequency of high-frequency keywords, and keywords with a frequency greater than or equal to 8 were selected as high-frequency keywords, a total of 30. The results are shown in Table 1.

**Table 1:** High frequency keywords.

No.	Keywords	Frequency	No.	Keywords	Frequency
1	Painting therapy	129	16	Children	13
2	Art therapy	121	17	Anxious	13
3	Painting art therapy	41	18	Quality of life	13
4	Schizophrenia	39	19	Self-respect	11
5	Fine art therapy	35	20	Group painting therapy	10
6	Expressive art therapy	32	21	Teenagers	10
7	University students	28	22	Special education	9
8	Mental health education	22	23	Self-efficacy	9
9	Autistic children	21	24	Emotional disorders	9
10	Depression	20	25	Datura painting therapy	9
11	Special children	19	26	Counseling	9
12	Group counseling	19	27	Psychological consulting	9
13	Psychotherapy	16	28	Art education	8
14	Recovery	15	29	Left-behind children	8
15	Fine art education	14	30	Application	8

As can be seen from Table 1, the top 10 high-frequency keywords all appear more frequently than or equal to 20, in the order of painting therapy (129), art therapy (121), painting art therapy (41), schizophrenia (39), fine art therapy (35), expressive art therapy (32), university students (28), mental health education (22), autistic children (21), and depression (20). The remaining 20 high-frequency keywords all appear more frequently than 8. The above results tentatively indicate that art therapy approaches such as drawing therapy have become a hot spot and focus of research in art therapy to help university students and special groups such as schizophrenics, autistic and depressed patients to carry out psychological treatment and rehabilitation. At the same time, art therapy methods such as group painting therapy and mandala drawing therapy are also receiving increasing attention from researchers in psychological counseling and consultation for other groups and fields.

### Similarity matrix analysis of Ochiai coefficients for high frequency keywords

The closer the value in the Ochiai coefficient similarity matrix is to 1, the closer the two keywords are to each other. And the more similar they are. Conversely, it indicates that the more distant the keywords are from each other, the less similar they are. The word-part matrix was imported into SPSS23 to generate the Ochiai similarity matrix for high-frequency keywords, and the results are shown in Table 2.

**Table 2:** Similarity matrix of Ochiai coefficients for high frequency keywords (parts).

	Painting therapy	Art therapy	Painting art therapy	Schizophrenia	Fine art therapy	Expressive art therapy	University students	Mental health education	Autistic children
Painting therapy	1.000	0.008	0.057	0.179	0.015	0.112	0.137	0.154	0.099
Art therapy	0.008	1.000	0.000	0.090	0.015	0.000	0.120	0.155	0.079
Paint art therapy	0.057	0.000	1.000	0.104	0.000	0.000	0.060	0.000	0.069
Schizophrenia	0.179	0.090	0.104	1.000	0.000	0.029	0.000	0.000	0.000
Fine art therapy	0.015	0.015	0.000	0.000	1.000	0.000	0.032	0.108	0.111
Expressive art therapy	0.112	0.000	0.000	0.029	0.000	1.000	0.067	0.151	0.000
University students	0.137	0.120	0.060	0.000	0.032	0.067	1.000	0.363	0.000
Mental health education	0.154	0.155	0.000	0.000	0.108	0.151	0.363	1.000	0.000
Autistic children	0.099	0.079	0.069	0.000	0.111	0.000	0.000	0.000	1.000

**Figure 1:**
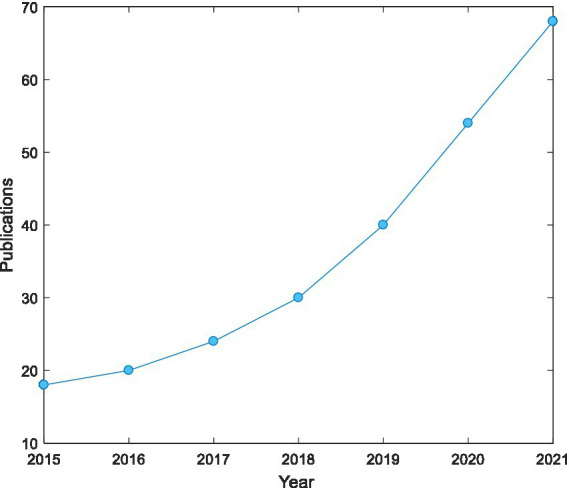
Number of publications on art therapy from 2015 to 2021.

As can be seen from Table 2, the various key words are in order of proximity to drawing therapy: schizophrenia (0.179), mental health education (0.154), university students (0.137), expressive art therapy (0.112), and children with autism (0.099), etc. This suggests that art therapy approaches based on drawing therapy, expressive art therapy and other art therapies have become an important part of art therapy in exploring art therapy for mental health education and treatment of different groups of people such as schizophrenics and university students. Figure 1 shows the number of Publications on art therapy from 2015 to 2021, it can be seen that this field is gradually attracting researchers’ attention.

### High frequency keyword clustering analysis

**Figure 2:**
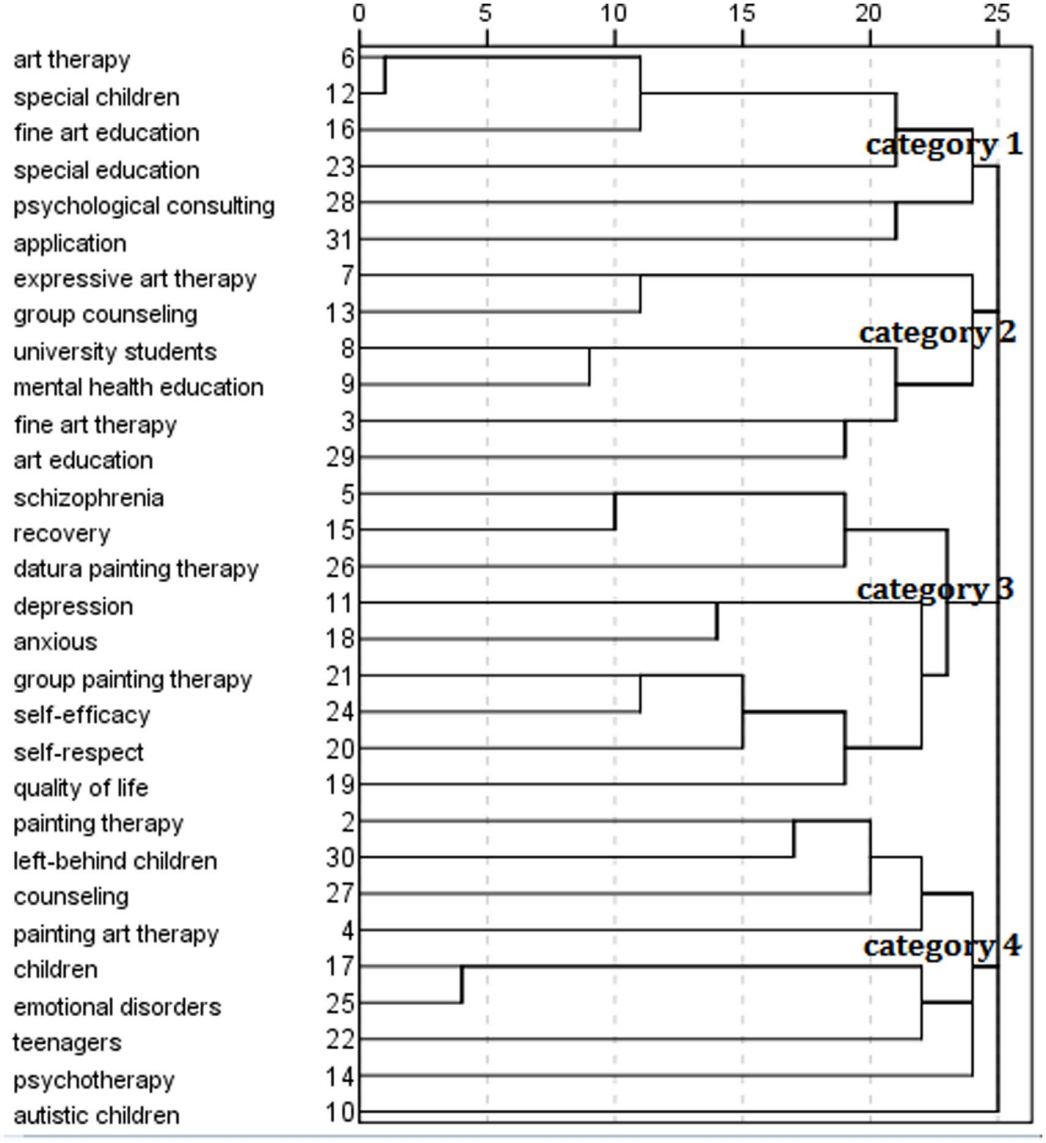
Cluster diagram of high–frequency keywords in art therapy research field.

In order to show the relationship between keywords more intuitively, the Bicomb 2.0 co-word analysis software was used to import the word-part matrix generated from 30 high-frequency keywords into SPSS23 for cluster analysis, generating a cluster analysis dendrogram of high-frequency keywords, as shown in Figure 2.

In Figure 2, the numbers on the vertical axis indicate the corresponding 30 keywords, and the numbers on the horizontal axis indicate the distance between the keywords. When the number is smaller, it indicates that the distance between the two keywords is smaller. When the similarity is higher, the closer the relationship is (Wang et al., 2021). According to the genealogical relationship of high-frequency keywords, they can be divided into four categories. Category 1 is the research on the application of art therapy in special children and their education, including six keywords of art therapy, special children, art education, special education, psychological consultation and application. Category 2 is the research on expressive art therapy in mental health education and group counseling of university students, including expressive art therapy, group counseling, college students, mental health education, art therapy, art education. Category 3 is a study of group painting therapy and mandala painting therapy in the rehabilitation of schizophrenia and depression patients, including 9 keywords such as schizophrenia, rehabilitation, mandala painting therapy, depression. Category 4 is a study of the psychological counseling and treatment of painting art therapy in different groups, including painting therapy, left-behind children, art education, and application. It includes nine keywords such as painting therapy, left-behind children and psychological counseling.

### Visual knowledge mapping for art therapy research

**Figure 3:**
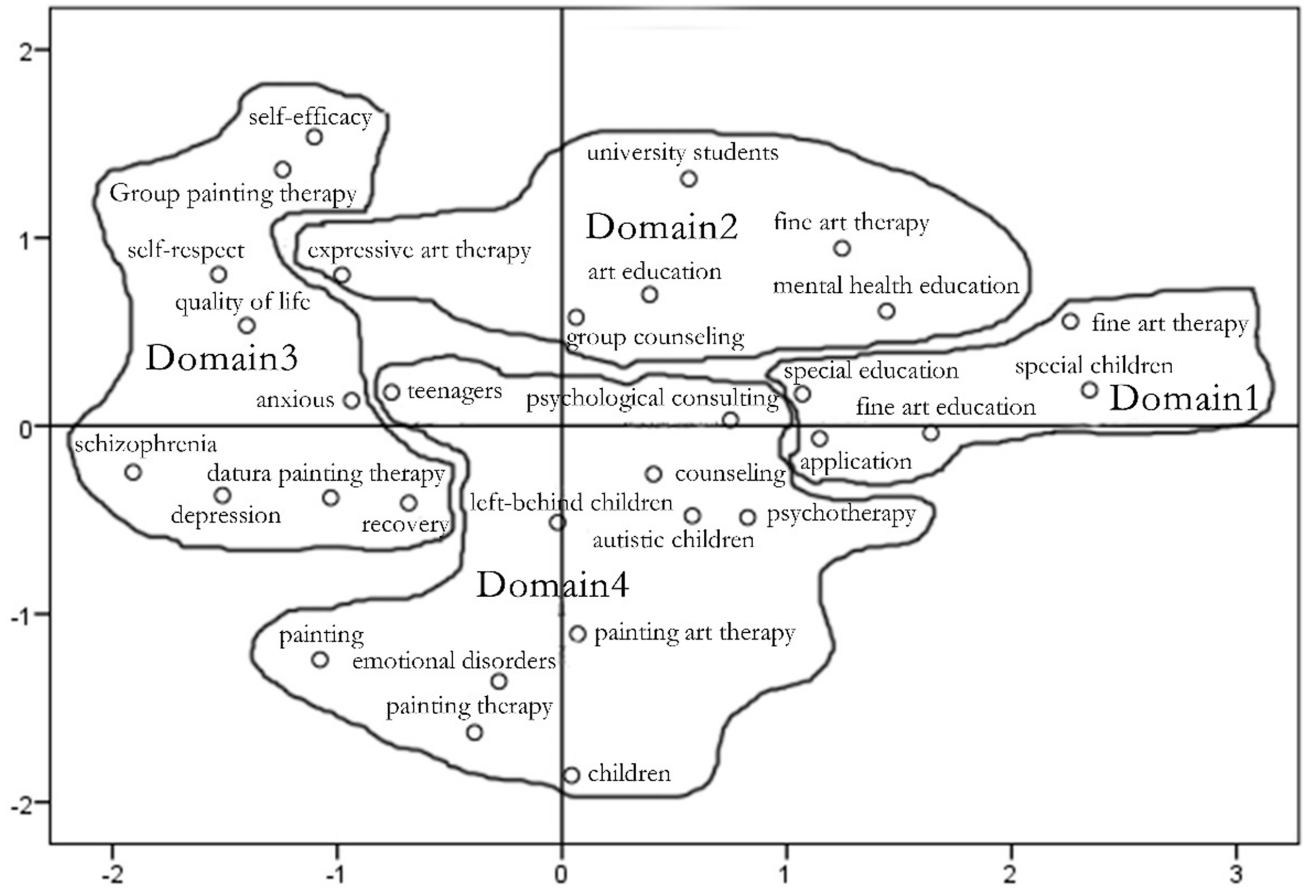
Knowledge map of hotspots in art therapy research.

The Ochiai similarity matrix was analyzed on a multidimensional scale using SPSS23 and combined with a tree diagram to create a visual knowledge map of hotspots in art therapy research, as shown in Figure 3.

In Figure 3, the smaller circles represent the position of the corresponding keywords. The closer the spatial distance between the circles, the closer the relationship is. Conversely, the relationship is more distant. The closer the circles are to the center of the strategic coordinates, the greater the influence of the keyword they represent. The horizontal axis of the coordinates represents centripetalism, the strength of inter-domain influence. The vertical axis represents density, the strength of inter-domain influence. As can be seen in Figure 3, the research on the application of art therapy to children with special needs and their education (corresponding to category 1) in domain 1 is mainly located in the first quadrant. In particular, “special children” and “special education” are located on the horizontal axis. This indicates that this area is a hotspot for research in this period and that there is a strong interaction between the areas. Area 2, expressive arts therapy in mental health education and group counseling for university students (corresponding to category 2), is located across quadrants 1 and 2. Among them, “expressive arts therapy” is in the second quadrant, “group counseling” is on the vertical axis, and the rest of the keywords are in the first quadrant. This suggests that there is good scope for the development of expressive arts therapy. There are strong interactions within this field and it is a hot spot for research at the moment. The research on group painting therapy and mandala drawing therapy for rehabilitation in schizophrenia and depression patients (corresponding to category three) in domain three is located in quadrants two and three. This suggests that group-based drawing therapy to enhance self-esteem, self-efficacy and quality of life in schizophrenia patients is of greater potential importance throughout the research and is constantly enriched. The thematic research area of mandala-based drawing therapy for the rehabilitation of depressed patients is internally well connected and has a clear title. However, it is marginal to the overall research. The research on counseling and treatment of painting art therapy in different groups (corresponding to category four) in domain four is mainly located in quadrants three and four. This indicates that the field is more tightly connected internally and that there is a body of research that has conducted formal research on it (Lavey-Khan and Reddick, 2020). However, it is also marginal to the overall research and has not yet received much attention.

## Trends in art therapy research

### The growing importance of art therapy in children with special needs and their education

In Figure 3, the keyword “art therapy,” which constitutes Domain 1, is in the first quadrant, while “special education” and “special children” are on the horizontal axis. This indicates that this area is a hot spot for research in this period and that there is a strong interaction between the areas. Art therapy is a form of therapy that integrates non-verbal expression, communication, communication and visual images. The art therapy method is applied to children with special needs. The application of art therapy to the education of children with special needs can help them to express their emotions through painting and to present their perceptions of their surroundings in a subjective way. As a “multi-focused intervention,” art therapy is often used for children with symptoms such as lack of self-awareness, inattention, difficulties in self-expression and rigid behavior (Harpazi et al., 2020). Lee and Liu (2016) attempted to find the improving effect of art therapy on behavioral adjustment and self-motivation of special educational children, they found that although the art therapy has little effect on sense of autonomy and their learning ability, art therapy can improve their behavioral adjustment and emotional *via* their parents’ reports. Art education is very important not only in general education, but also in special education. The integration of art therapy into special and general education to promote special students’ skills, emotional regulation and self-expression has recently become more and more important (Allahverdiyev et al., 2017). For autism treatment, research has shown that the use of art therapy can help people with autism improve their social skills (Yücesoy et al., 2020). In additional, another research also pointed out that art therapy provides a non-verbal language advantage with effect of strong feeling, which can be used for special children treatment (Petruta-Maria, 2015). In summary (Srolovitz et al., 2022), it can be found that art therapy can effectively improve the physical and psychological problem behaviors of children with special needs, which makes it increasingly important in special education in China.

### Expressive arts therapy has good prospects for development in mental health education and group counseling for university students.

In Figure 3, the keywords “art therapy,” “art education,” “mental health education” and “university students” are all located in the first quadrant. This indicates that this area is also a hotspot and focus of research, with strong interactions within the area and that expressive arts therapy has good potential for development. Expressive arts therapy originates from art therapy, a counseling and therapeutic approach that uses and integrates different art forms in psychotherapy to help clients express their inner emotions and improve their personality (Otting and Prosek, 2016). It generally consists of two working orientations, namely the art psychotherapy orientation and the art therapy orientation (Stuckey and Nobel, 2010). The former uses art as a medium of non-verbal communication to help visitors understand and resolve their emotional problems through artworks that express the inner world, together with verbal associations and explanations, focusing on non-verbal communication and interpretation between the therapist and the visitor in the process of art creation. The latter is a process of artistic expression that uses the power of artistic activity to alleviate the individual’s internal conflicts and conflicts with the social environment, to raise awareness, to give vent to emotions and to sublimate emotions, and is more artistic in nature. Expressive arts therapy is an intuitive way of thinking that allows for the timely expression of repressed content in the visitor’s subconscious in a non-verbal, symbolic manner. It also allows for a sense of safety, less impediment and facilitates the gathering of authentic information. It can be applied to a wide range of clients, breaking through the limitations of different visitors’ ages, languages, cognitive ranges and artistic skills, which is highly flexible and versatile. It is also a safe way to release destructive energy, such as anger and hostility, in a way that is socially acceptable (Maiese, 2016). Expressive arts therapy is now widely used in individual counseling and group counseling in colleges and universities. College counselors use professional working environments, such as counseling rooms, drawing therapy rooms, integrated group therapy rooms and other separate spaces to provide a comfortable and reassuring environment for students to express themselves. Based on the two working orientations of expressive arts therapy, counselors use these techniques to focus on the inner state projected by the visitor from therapy, using the work created as a tool to detect psychological states, and to guide the visitor to experience self-growth in expressive arts therapy. The integration of expressive arts therapy techniques into mental health education programs is an important way to promote reform of mental health programs in universities. Using the strong experiential sense of art to guide students to deeply engage with the theme of mental health education teaching not only increases student participation and leads to spontaneous thinking, but also enhances the fun of the classroom and increases student motivation. In addition, most cultural activities on campus are themed around creative artistic expression. The flexible use of expressive art therapy in campus activities can create a harmonious campus atmosphere through enriching activities, while helping students to present their problems in interpersonal relationships, academic stress and self-growth confusion through artistic creation, providing a platform for their psychological growth (Torgerson, 2018). Therefore, the therapy has good prospects for development in mental health education and group counseling for university students.

### Group painting therapy continues to be enriched

In Figure 3, the key words “self-efficacy,” “group drawing therapy,” “self-esteem” and “quality of life,” which make up Domain 3, are located in the second quadrant. The research subjects of this domain, “schizophrenia” and “depression,” are located in the third quadrant. This indicates the potential importance of group drawing therapy in the overall study. However, research on people with schizophrenia and depression is still marginal. Schizophrenia is a complex mental illness that severely impairs life and social functioning, and one of its core features is cognitive deficits (Li, 2020). Patients with schizophrenia show varying degrees of abnormalities in personality traits, attention, memory, processing speed, executive functioning, language expression, thought perception, spatial ability and social cognitive ability (Wu et al., 2017). Depression is a common psychiatric disorder characterized by low mood and a lack of pleasure (Karyotaki et al., 2021), in which altered cognitive functioning is not only a typical symptom of depression, but also an important risk factor for depressive episodes (Mirza et al., 2017). Group painting therapy is a form of psychotherapy that uses drawing as a mediator and a group approach (Yuan et al., 2021). It allows patients to use non-verbal tools to reveal subconsciously repressed feelings and conflicts in the process of drawing, which can be expected to be uniquely useful for people with schizophrenia and depression who have emotional, cognitive and social impairments. The non-verbal imagery of drawing can also be used to avoid consciousness, defense mechanisms and impediments to a greater extent, enabling individuals to express a richer experience of psychological content and compensating for the lack of interview-based psychotherapy (Simon and Kovács, 2015). In addition, compared to individual drawing therapy, group painting therapy provides multiple perspectives on the experiences and feelings of others, enhances the ability to actively develop oneself (Lavey-Khan and Reddick, 2020). It also allows patients to enhance their creativity, promote emotional and cognitive recovery, improve social functioning and enhance their quality of life. The treatment has been widely used in developed countries such as Europe and the US (Joschko et al., 2022), which is generally recognized for its effectiveness in the rehabilitation of patients with schizophrenia and depression. Domestic studies on their group painting therapy have also found that group painting therapy helps to improve the symptoms of patients with schizophrenia and promotes the recovery of their self-esteem and self-efficacy (Yuan et al., 2021). It is also effective in improving the cognitive functioning of depressed patients, increasing their self-esteem levels, improving their sleep quality and reducing their negative emotions (Rointan et al., 2021). In summary, it can be found that the connotations in group painting therapy regarding schizophrenia and depression patients are constantly enriched, from the cognitive and emotional problems caused by the individual’s physiology to a greater focus on their inner psychological enhancement such as self-esteem, self-efficacy and quality of life.

### Research on the subject of mandala painting therapy is closely related, but research is marginal.

In Figure 3, the keywords “mandala painting therapy,” “schizophrenia,” “anxiety,” “depression” and “recovery,” which make up Domain 3, are all located in Quadrant 3. This indicates that the field is well connected and has a clear title, but is marginal to the overall research. Mandala painting therapy, developed by Carl Jung, the founder of the psychoanalytic school, refers to a non-verbal psychotherapy based on the principles of projection, expression, symbolism, sublimation and externalization, mediated by mandala painting tools, in which individuals paint mandalas in order to present the repressed content of their personality and subconscious and achieve catharsis, emotional improvement, trauma repair and personality integration in the process of painting (Kostyunina and Drozdikova-Zaripova, 2016). It is a widely used form of psychological assessment and therapeutic drawing psychotherapy, which has the function of projecting the inner world and healing the mind through drawing, reducing psychological disorders and maintaining inner order (Angellim et al., 2020). Mandala painting includes both structural and non-structural forms, structural mandalas where the painter colors in a pre-designed mandala pattern and non-structural mandalas where the painter paints freely within the required blank circle (Lee and Kim, 2020). In current research, mandala painting therapy is mainly applied to the rehabilitation of anxiety and other emotions in schizophrenia and depression patients. It has been found that mandala painting therapy can effectively promote the alleviation of anxiety, improve mood, enhance their self-esteem, improve interpersonal and communication skills, and thus promote the recovery of their social functions in schizophrenia patients in recovery (Cox and Cohen, 2000). Mandala graphics present a cosmic outlook, and the process of painting helps to stabilize mood, alleviate depression and stimulate personal potential. Self-intervention in depressive states through mandala painting therapy helps depressed patients to regain their courage and self-confidence to overcome their illness and to regain their initiative in life, thus becoming more proactive in participating in functional training (Petrishscheva and Filatova, 2017). In addition, as a result of the active therapeutic intervention, the patient’s neurological function is restored, the ability to perform daily activities is significantly improved, and the depressive symptoms are correspondingly improved, thus reinforcing the patient’s confidence that he or she can overcome the illness, thus reaching a virtuous circle (Liu and Lu, 2018). However, research on mandala painting therapy is still marginal, probably due to the fact that there are fewer psychologists and related researchers specializing in mandala painting therapy, which has led to less research in this area and consequently to the marginalization of research in this field.

### The focus of painting art therapy on the study of counseling for different groups of people has not received much attention in the overall study

In Figure 3, the keywords “painting art therapy,” “emotional disorders” and “left-behind children,” which constitute domain 4, are all located in the third quadrant. The first two keywords are close to each other, indicating that they are closely related to each other. The keywords “psychological counseling,” “psychotherapy,” “psychological counseling” and “autistic children” are all located in the fourth quadrant. The fact that they are in the fourth quadrant indicates that the field is well connected and has been formally studied by research institutions, but is also marginal to the overall research and has not received much attention. Mood disorders in children refer to a psychological disorder that occurs in childhood or adolescence, with anxiety, fear, depression and obsessions as the main clinical manifestations (Huang et al., 2012), which seriously affects the normal physical and mental health of children. In the past, children with mood disorders were often treated with medication, but the results were not satisfactory and the incidence of adverse effects in children was high. Art therapy is a non-verbal way of expressing and communicating with the child, in which the child is encouraged to express his or her emotions and bring out his or her subconscious emotions and inner conflicts. It is a form of psychotherapy that is closer to nature and relies on the individual to feel the environment. As a common form of art therapy, painting can not only help individuals to resolve or alleviate emotional problems or psychological disorders caused by psychological factors, but can also help clients to integrate their feelings, improve their self-understanding, promote growth and provide a comfortable and pleasant emotional experience through the process of painting (Bitton and Laufer, 2018). It has been found that children with emotional disorders can learn to re-conceptualize themselves, self-reflect and think through the process of drawing, which can also use drawing to vent their inner emotions and release their inner repression, thus facilitating their emotional expression, alleviating emotional disorders and changing their behavioral patterns (Paine et al., 2021). At the same time, the therapist can judge the child’s psychological condition through the content of his or her drawings and provide him or her with targeted psychological guidance to enable him or her to better express his or her hidden emotions (Jalambadani, 2020). It has also been found that art therapy is beneficial to the emotional and cognitive development of children with autism, improving their social and verbal skills and enhancing their rehabilitation outcomes (Sampurno et al., 2020). For children left behind who are introverted, withdrawn, self-conscious and unsociable, drawing art therapy can also create a harmonious and calm psychological environment for them, enhance their self-confidence and self-esteem, and help them to vent their emotions and build good interpersonal relationships (Zhang et al., 2021). The art of drawing is also used to create a harmonious and calm psychological environment for children, enhance their confidence and self-esteem, and help them to vent their emotions and build good relationships. This shows that pictorial art therapy focuses on counseling and therapy for different groups of people to improve their psychological problems. However, this area has not received much attention in the overall research. Therefore, further research is needed in this area in future studies.

## Conclusion

To explore the relationship between art therapy and special children, through the analysis of the knowledge map of research hotspots of art therapy in China’s education since the 21st century, it is found that art therapy is increasingly valued in special children and their education. Expressive art therapy has good prospects for development in the mental health education and group counseling of university students. The connotation of group painting therapy is constantly enriched; mandala painting therapy is closely related to thematic research, and painting art therapy focuses on the study of psychological counseling for different groups, but these two are still on the periphery of the overall research and have not yet attracted attention. Since the 21st century, the research progress of art therapy in education in China has made certain achievements. However, future research should also focus on the following areas. Firstly, continue to strengthen the research on art therapy and expressive art therapy in special education and individual mental health education. Secondly, the depth and breadth of group drawing therapy should be further broadened. Thirdly, research into mandala painting therapy and painting art therapy needs to be increased.

## Author contributions

YC data analyzes and writing of the manuscript. FW critical review of the manuscript. All authors contributed to the article and approved the submitted version.

## Conflict of interest

The authors declare that the research was conducted in the absence of any commercial or financial relationships that could be construed as a potential conflict of interest.

## Publisher’s note

All claims expressed in this article are solely those of the authors and do not necessarily represent those of their affiliated organizations, or those of the publisher, the editors and the reviewers. Any product that may be evaluated in this article, or claim that may be made by its manufacturer, is not guaranteed or endorsed by the publisher.
